# Association between Working Time and Burnout Syndrome in Peruvian Military during the Second Epidemic Wave of COVID-19

**DOI:** 10.3390/ijerph192013614

**Published:** 2022-10-20

**Authors:** Mario J. Valladares-Garrido, Luis Eduardo Zapata-Castro, Cinthia Karina Picón-Reategui, Ana Paula Mesta-Pintado, Ronald Alberto Picón-Reategui, Mariana Huaman-Garcia, César Johan Pereira-Victorio, Danai Valladares-Garrido, Virgilio E. Failoc-Rojas

**Affiliations:** 1 South American Center for Education and Research in Public Health, Universidad Norbert Wiener, Lima 15046, Peru; 2 Oficina de Epidemiología, Hospital Regional Lambayeque, Chiclayo 14012, Peru; 3 Escuela de Medicina, Universidad Nacional de Piura, Piura 20002, Peru; 4 Scientific Society of Medical Students, Universidad Nacional de Piura, Piura 20002, Peru; 5 Escuela de Medicina, Universidad de San Martín de Porres, Chiclayo 15011, Peru; 6 Escuela de Medicina, Universidad Cesar Vallejo, Piura 13001, Peru; 7 School of Medicine, Universidad Continental, Lima 15046, Peru; 8 Unidad de Epidemiología y Salud Ambiental, Hospital de Apoyo II Santa Rosa, Piura 20008, Peru; 9 Research Unit for Generation and Synthesis Evidence in Health, Universidad San Ignacio de Loyola, Lima 15024, Peru

**Keywords:** burnout, stress, mental health, impact of the COVID-19 pandemic, military, Peru

## Abstract

There is scant evidence on the impact of the COVID-19 pandemic on burnout in front-line military personnel and how working time may influence on this condition. We aimed to determine the association between working time and Burnout syndrome in military personnel. A cross-sectional study was conducted using secondary data among 576 military personnel from Lambayeque, Peru during the second wave of COVID-19 in 2021. We used the Maslach Burnout Inventory instrument to measure Burnout Syndrome. We evaluated its association with work time, measured as the number of months that the military member worked during the pandemic. The prevalence of burnout syndrome was 9%. Of the total sample, 39.1% and 10.3% presented depersonalization and emotional exhaustion, respectively. Military personnel working for more than 18 months had a 104% higher prevalence of Burnout syndrome (PR: 2.04, 95%CI: 1.02–4.10). Exposure to a prolonged work time during the pandemic increased the prevalence of Burnout syndrome in military personnel. This information helps to understand the potential effects of the pandemic on this population and provides insight into the time the military members would need rest to prevent Burnout syndrome.

## 1. Introduction

The COVID-19 pandemic has had a major impact on mental health worldwide [1,2,3,4,5,6,7]. Burnout Syndrome (BS) is a mental health problem caused by overwork or stress and affects different types of workers [8,9,10]. During the COVID-19 pandemic, health, police, and military personnel led the frontline teams, so they were exposed to a high risk of direct contagion with people with suspected COVID-19. Military personnel have carried out multiple activities to ensure the safety, mobility, social distancing, and immunization of the population against COVID; they have provided support to health entities (Grupo Comando COVID-19 Sipán-Lambayeque Perú), food distribution, transfer of sick and dead people as a result of COVID-19, among others. [11,12]. For this reason, there has been an increase in cases of BS in the military, but also of emotional alterations, depersonalization disorder, and negative impact on aspects of their fulfillment [13].

Of Mexican policemen, it was found that 39.1% presented emotional exhaustion [14]. In another study, 32.9% of BS was estimated in a group of military personnel during the COVID-19 pandemic [15]. Likewise, at the beginning of the pandemic, a higher frequency of BS was reported in the military compared to the civilian population [15]. Furthermore, in another study conducted on military physicians in the United States, it was found that BS and emotional exhaustion affected 10% and 73%, respectively [16]. While in Brazilian police officers, a higher frequency of Burnout symptoms was reported in those who consumed alcohol and drugs, considering as possible risk factors [9]. Other multiple risk factors have been documented for the development of BS in the military: stress, time spent at work, and not having time to spend with family and friends, among others [13,14,15]. Conversely, some important protective factors for BS are physical activity, religious affiliation, personal self-realization, and job gratification [14,17], although there might be many others.

Multiple studies have evaluated the impact of COVID-19 on mental health [6,18,19]. However, most have focused on healthcare professionals, the general population, and even university students [20,21,22,23]. Additionally, there is inconclusive evidence on the effect of the pandemic on the development of emotional and/or physical exhaustion in the military [24]. Furthermore, previous studies have not measured potential influencing factors for the development of BS: having chronic diseases (arterial hypertension, obesity) [25] and seeking psychological help during the pandemic [14], which have been addressed in this research. Neither has the perception of military personnel regarding the government’s actions during the COVID-19 health emergency been analyzed [26,27], which would be considered as a possible factor observed by this type of population regarding the commitment of the authorities to support their workers. Our research is relevant because it is necessary to document solid evidence of the impact of COVID-19 on military burnout. In addition, information bias was present in previous research [28,29]. Another point not considered in similar studies on military workers is the lack of evaluation of circumstances such as the experience of post-traumatic stress since they work in dangerous situations and face critical incidents that can be potentially traumatic [14,29]. Additionally, other studies lack rigorous biostatistical methods and only focus on descriptive results [17,25,28].

Workload has been shown to affect mental health in the military. However, it is unknown how much the pandemic has affected this phenomenon. We propose that time on duty during this stage would entail prolonged time without leisure or family activities, which may increase the risk of BS. Therefore, this study aimed to identify whether working time is associated with BS in the Peruvian military personnel. For this purpose, we have proposed the following hypothesis: (1) The prevalence of BS in the Peruvian military personnel is high during the pandemic, (2) There are specific characteristics in this population that increases the prevalence of BS, and (3) higher time in service is independently associated with the development of BS.

## 2. Materials and Methods

### 2.1. Study Design and Population

An analytical cross-sectional study of secondary data analysis was carried out, whose objective was to evaluate whether working time influences the presence of Burnout Syndrome in military personnel in the Lambayeque region, Peru from 2 to 9 November 2021.

The population consisted of 820 military personnel who were working in the first line of attention in front of COVID-19 in the Lambayeque region, Peru during the second wave (2021). In the primary study, a sample size of 582 individuals was estimated using an expected prevalence of 12.8% [30], 99% confidence level, precision of 2.5%, and adding 20% of the sample to compensate for possible participant losses and rejections. A higher number of participants (n = 710) than required was obtained, representing 86.6% of the study population.

For the present analysis, records from 576 military personnel were used after excluding 134 observations with missing information of the Maslach Burnout Inventory (MBI), which measured the dependent variable. A convenience sampling method was used.

### 2.2. Procedure

First, permission was requested from Lambayeque military authorities. Then, the principal investigator trained the field team to conduct the interviews, under the supervision of the military officer and designed the questionnaires in REDCap, a data entry system with rigorous data collection, validation, and quality control characteristics. Interviews were conducted with military personnel in person using REDCap. A link to the questionnaire was created in REDCap and shared with the supervising military officer. The military population was organized into three groups in two daily shifts (morning and afternoon). Once the military was gathered, the officer in charge distributed the link via text message and/or WhatsApp. The military members then filled out the informed consent form and subsequently answered the questionnaires of interest from their phones. The field team was attentive to resolving doubts and technical problems that could arise at the time of filling out the interviews with the military. At all times, compliance with biosecurity measures was verified: correct use of masks, social distancing, use of alcohol gel for hand disinfection, and use of open spaces in military centers.

### 2.3. Measures

The dependent variable was BS, which was measured with the Maslach Burnout Inventory-Human Services Survey (MBI-HSS). The Spanish-validated version of the instrument was used [31,32]. It is composed of 22 questions divided into three dimensions, which uses a Likert scale from 0 (never) to 6 (every day): 9 emotional exhaustion (EE) questions, 5 depersonalization (DP) questions, and 8 personal realization (PR) questions. BS was operationally defined as military personnel who presented low levels of PR and high levels in the DP and EE dimensions [33]. We used tercile classification of each dimension, where values above the 66th percentile were considered high and those below the 33rd percentile as low. The MBI-HSS presents excellent psychometric properties of validity and reliability (comparative fit index-CFI: 0.91) [31,34]. In addition, high internal consistency has been estimated in each dimension: EE (Cronbach’s alpha: 0.84), DP (Cronbach’s alpha: 0.71) and PR (Cronbach’s alpha: 0.75) [35]. This instrument has been used in the military population of the Peruvian army [28,36,37]. In our research, we found optimal internal consistency. The overall Cronbach’s alpha was 0.91. In the dimensions of EE, DP, and PR we estimated a Cronbach’s alpha value of 0.90, 0.76, and 0.93, respectively.

The main independent variable was working time, operationally defined as the report of working time since the onset of the COVID-19 pandemic in months. This variable was categorized as 1 to 6 months, 7 to 12 months, 13 to 18 months, and 19 months or more.

Fear of COVID-19 was measured with the Fear of COVID-19 Scale (FCV-19S). This questionnaire consists of 7 questions and uses a Likert scale from 1 (strongly disagree) to 5 (strongly agree). It presents excellent levels of validity (test–retest reliability: 0.72) and reliability (Cronbach’s alpha: 0.72) in the general population [38].

The level of physical activity was assessed with the International Physical Activity Questionnaire, short version (IPAQ-S). This instrument collects self-reported information of the level of physical activity that the participant performed during the last 7 days through 4 domains (leisure time, transportation, work, and domestic activities) [39]. The short version is composed of 9 items. It presents adequate reliability and validity. It has been applied in the Latin American population [40,41] and during the COVID-19 pandemic [42].

General-psychosocial and other data section were the following: general variables were measured (age, sex, marital status, religion, having a child), report of pathological antecedents (harmful habits, body mass index, arterial hypertension), psychosocial (food insecurity, report of having sought help for mental health problems, trust in the government to manage the pandemic).

### 2.4. Statistical Analysis

Stata 17 was used for the data analysis.

Frequencies and percentages were estimated for descriptive analysis of categorical variables. For numerical variables, normal distribution was evaluated and then the best measure of central tendency and dispersion was estimated.

In the bivariate analysis, the chi-square test was used to evaluate the association of the independent variables for BS. For numerical variables, the nonparametric Mann–Whitney U test was used. A significance level of 5% was used.

To estimate whether working time and other covariates are associated with BS, prevalence ratios (PR) and confidence intervals (95%) were estimated using generalized linear models (GLM) with Poisson distribution, log link function, and robust variance. This technique allows analyzing dichotomous values of the outcome (No BS = 0, BS = 1), transforming the original scale into a logarithmic scale and increasing statistical precision by excluding outliers. The association of interest (working time and BS) was adjusted for the secondary independent variables. Potential collinearity between the independent variables included in the multiple regression model was evaluated with the variance inflation factor (VIF) test. All the variables entered the model since collinearity was not significant.

### 2.5. Ethical Aspects

The primary study was approved by the Ethics Committee of Universidad San Martin de Porres with document number 269-2022-CIEI-FMH-USMP. The confidentiality of the participants was maintained, and informed consent was requested from each military member. The database obtained for the analysis was anonymized.

## 3. Results

We analyzed a sample of 576 military personnel between 2 to 9 November 2021. The characteristics of participants are shown in Table 1.

### 3.1. Work Time and Other Factors Associated with Burnout Syndrome in Bivariate Analysis

We found statistically significant differences in the bivariate exploratory analysis between the prevalence of BS and the variables physical activity (*p* = 0.021) and work time (*p* = 0.006). Table 2 details the bivariate analysis.

### 3.2. Work Time and Other Factors Associated with Burnout Syndrome in Simple and Multiple Regression Analysis

In the simple regression model, we found that military personnel who had been working for 19 months or more during the COVID-19 pandemic had a 94% higher prevalence of presenting BS (PR: 1.94; 95%CI: 1.01–3.75). This association was maintained in the multiple regression, where the prevalence of BS increased by 104% in military personnel with that range of working time (19 months or more) (PR: 2.04, 95%CI: 1.02–4.10). Additionally, we found that divorced military personnel had a higher prevalence of BS than single military personnel (PR: 8.08; 95%CI: 2.05–31.82) (Table 3).

In the analysis of BS dimensions, we found that military personnel with working time longer than 18 months had a 30% lower prevalence of emotional exhaustion (PR: 0.46, 95%CI: 0.22–0.97). No statistically significant difference was observed between work time and the depersonalization and personal realization dimensions (Table 3).

Obese military personnel (PR: 3.21; 95%CI: 1.17–8.80) and with fear of COVID-19 (PR: 5.71; 95%CI: 3.43–9.0) had a higher prevalence of emotional exhaustion. In contrast, military personnel with non-Catholic religion were associated with a lower prevalence of this dimension (PR: 0.41; 95%CI: 0.17–0.98) (Table 3).

The prevalence of depersonalization increased in military members with food insecurity (PR: 1.29; 95%CI: 1.03–1.61) and fear of COVID-19 (PR: 2.03; 95%CI: 1.66–2.48). In the case of the personal fulfillment dimension, we found that military cohabiting (PR: 1.82; 95%CI: 1.13–2.94), Catholic (PR: 1.55; 95%CI: 1.08–2.22), high physical activity (PR: 2.24; 95%CI: 1.34–3.73) were positively associated with this dimension (Table 3).

## 4. Discussion

### 4.1. Prevalence of Burnout Syndrome

The prevalence of BS was 9% [43]. This is almost similar to that reported by Queirós C. et al. who documented a prevalence of 11% in police officers in a literature review covering 26 countries worldwide [29]. However, it differs from the findings in specific European countries such as Spain 28.5–43.6% and Germany 46.6% in police officers [11,14,43]. These heterogeneous findings could be explained by two reasons; first, by a difference between the instruments used to assess BS, among them the Spanish Burnout Inventory (SBI) and the Maslach Burnout Inventory (MBI) [44,45]. Secondly, there is a difference in the appearance of the SARS-CoV-2 waves, in Europe, the second wave was from 2020 to January 2021; while in Peru and Latin America it arrived later between January 2021–August 2021, which allowed more time for emergency preparedness and response [44,45]. Nonetheless, it is vital to emphasize that these data would be justified by the highly stressful workload of such personnel [11] since among their main activities would be to provide security in support-contingency facilities, preserve internal order with compliance with schedules during curfews and mandatory social immobilization, control social distancing in crowded places; as well as the transfer of patients to isolation zones and home visits in areas of very high epidemiological risk for COVID-19, which would explain the appearance of BS [46,47]. Additionally, added to the surveillance orders, order control, restrictions, economic consequences, limited resources in our health system, and a large number of deaths, created adverse exposures both at home and at work for military personnel, who have been deployed in the country to support pandemic response efforts [48].

In addition, we found that 1 in 10 military personnel experienced emotional exhaustion, and 39.1% experienced depersonalization. Emotional exhaustion is defined as a state in which an individual feels emotionally worn out and drained because of accumulated stress in one’s personal or work life. Depersonalization is defined as a distant or indifferent attitude towards work, which manifests itself as insensitive and cynical behavior. The result found in this study is higher than that reported by a study in Mexico where 30.7% were found in the military [15]. Additionally, it differs from that found in European studies with a prevalence of 58.0% [11]. This could be explained by the fact that military personnel is a different population group since they are constantly mediating agents in situations characterized by serious conflicts and tensions [9]. They stand out among other professionals due to their high level of stress, causing untimely physical-emotional exhaustion [9] which, added to the responsibility of making difficult decisions, together with concerns about their exposure and that of their family, generates a negative impact on mental health in the short and long term, especially because depersonalization can lead them to a loss of motivation with a feeling of isolation, in an environment of maximum responsibility [49].

### 4.2. Working Time and Burnout Syndrome

Working 19 months or more in the face of the COVID-19 pandemic almost double the prevalence of BS. To our knowledge, there is no documented evidence on military personnel. Some studies revealed that the COVID-19 pandemic increases the prevalence of burnout in health professionals by up to 80%, especially in countries with a high number of COVID-19 infections [50]. This finding could be explained given that our first line of COVID-19 containment, which includes military personnel, was exposed to high job strain for a longer time relative to other groups, including working in stressful situations due to constantly changing control measures, deployment to new environments and high-risk areas, resource shortfalls, and fear of transmitting infection [51]. However, the prevalence of emotional exhaustion is reduced to 54% in military personnel working 19 months or more. This differs from what is found in the literature, since there are few documented reports with the same amount of time that our study evaluated (e.g., a 40.9% prevalence of burnout at 15 months of work activity has been reported [52]). This finding could be explained by reduced emotional exhaustion during the study period since there was already a first vaccine coverage for frontline staff.

### 4.3. Factors Associated with Burnout Syndrome

In our research, we found that having a divorced marital status increases the prevalence of burnout syndrome. Currently, there is a knowledge gap regarding specific studies with burnout. Similar mental health outcomes have been reported with other outcomes such as being at the lowest level for mental functioning at 14.30% in divorced military personnel [53]. This is similar to that reported in the world literature, as divorce consistently ranks among the most stressful life events in adulthood [54]. Even 20% of divorcees experience pronounced psychological problems and lower well-being even years after their separation, in addition to being associated with core components of burnout such as stress, distress, decreased coping, and disengagement [54]. This finding may be justified given that the association with adverse mental health outcomes including the emotional burden of divorce and chronic stress, along with the increased likelihood of subsequent military deployment, has been robustly demonstrated [53].

Obesity increased the prevalence of emotional exhaustion by 221%. This is similar to that reported by Kress A. et al. who have reported that in military personnel obesity increased the prevalence by 33% and 240% among men and women, respectively, [25]. Despite this finding, there is no additional evidence in our group of interest. The medical literature indicates that there is reverse causality between obesity and other mental health problems [55]. Two meta-analyses reported positive associations between obesity and anxiety-depression disorders (OR: 1.40 and OR: 1.58 respectively) [56,57]. Another meta-analysis reported that obesity increases the prevalence of depression by 55% (pooled OR: 1.55) [55]. Additionally, it has been found that obese workers have a higher risk of emotional exhaustion than those with a healthy weight (OR: 1.28) [55].

Regarding depersonalization, we found that military personnel with food insecurity had a 29% higher prevalence of depersonalization. Food insecurity is defined as the condition of not having reliable access to enough affordable and nutritious foods. So far, there are no solid reports of this association in this study group. For example, Beymer M. et al. found that 32.8% of military personnel had food insecurity and that this was significantly related to other mental health outcomes such as anxiety, depression, and suicidal ideation (OR:1.82, OR:1.78, OR:1.78; respectively) associated in turn to leave the service [58]. This result suggests that food insecurity may influence diverse dimensions in mental health, including burnout.

In addition, military personnel with fear of COVID-19 significantly increased the prevalence of emotional exhaustion. This is similar to that reported by Martinez-Cuazitl et al. who reported 32.9% emotional exhaustion in military personnel [15]. Additionally, other studies found 35.7% with moderate and 31.9% with severe emotional exhaustion, being the main predictors of this condition the working hours, psychological comorbidities, and fear of infection [59]. This finding could be explained because fear is a key source of mental health problems related to anxiety and distress [60], especially in an environment where our military personnel seeks to comply with the provisions of the Peruvian government, where there are high rates of infection given that 50,616 confirmed cases and 787 deaths have been recorded in the police; while in military personnel a total of 2000 and 70, respectively, surpassing any other professional group in Peru [61,62].

Regarding personal fulfillment, we found that military personnel with a high level of physical activity increased the prevalence of personal fulfillment by 124%. This association is not documented in the literature with our population of interest. However, it has been described that physical activity influences well-being and personal realization, as well as pain avoidance [63]. This association could be explained by the fact that military personnel with a high level of physical activity probably generate significantly higher levels of life satisfaction, self-esteem, and happiness compared to inactive people, especially because the biological pathways of physical activity act as a buffer against stress and stress-related disorders, optimize neuroendocrine and physiological responses to stressors and promote an anti-inflammatory system, improving neuroplasticity along with growth factor expression [64], and even generate lower levels of anxiety; thus improving their self-efficacy and motivation during daily life [65,66].

### 4.4. Implications of Findings in Mental Health

Attention should be paid to this population group and their mental health treatment and prevention strategies, since in a context of a COVID-19 pandemic, added to other possible risk factors such as substance abuse and constant stress due to intense work schedules in the first line of containment, could worsen the panorama. Especially since it has been shown that mental health stigma still exists among military personnel [15], it is vital to reduce the gaps in care in this area of health. Additionally, these findings are important for the design of better mental health wellness interventions and policies that involve physical activity with great support for military personnel and their families during emergencies.

### 4.5. Limitations and Strengths

The main limitations of our study are firstly a possible measurement bias given that variables such as stress, level of job satisfaction, coping styles, and social support could not be assessed which could have influenced our BS outcome [67]. Second, there is a possible selection bias given that we cannot infer that our study had a large coverage of all military personnel, as we only had one study site. Finally, given our cross-sectional study design, it does not allow us to have the necessary temporality to the data collected to attribute causality.

Nevertheless, our study relies on the use of properly validated instruments, adapted to our reality and with optimal psychometric properties to assess the main mental health outcomes. Additionally, our research includes coverage of military personnel in a department severely hit by the COVID-19 pandemic in Peru, thus helping to measure the urgent need to implement better community mental health services needed for different population groups that are destined to preserve order and security as the first line of containment of the COVID-19 pandemic [47]. Under this scenario, the mental health of military personnel is a public health problem that needs to be addressed, our study shows solid and documented evidence to give rise to future follow-up studies in military personnel in the northern region of Peru.

## 5. Conclusions

This study evidenced that roughly nine out of 100 Peruvian military personnel experienced BS during the pandemic. In particular, working time was a significative factor that influenced this condition. Other factors associated with increased prevalence of BS were having a divorced marital status, fear of COVID-19, obesity, food insecurity, and a high level of physical activity. These findings suggest that some individuals need special mental health attention to prevent BS and other mental health outcomes. With this information, solid and first-hand evidence are provided to contribute to the development of better policies and better-implemented interventions to strengthen mental health prevention and control, especially during a national emergency context. For example, there may be workshops fostering physical exercise, improving conditions for personnel with families, and identifying and approaching individuals with multiple risk factors. Based on our results, a service time of less than 19 months may be necessary to provide sufficient rest and prevent BS.

## Figures and Tables

**Table 1:** Characteristics of participants.

Characteristics		N (%)
Age (years) *		22 (19–32)
Gender		
	Female	31 (5.4)
	Male	545 (94.6)
Marital status		
	Single	420 (72.9)
	Married	136 (23.6)
	Cohabitant	13 (2.3)
	Divorced	7 (1.2)
Religion		
	None	83 (14.4)
	Catholic	401 (69.6)
	Non-Catholic	92 (16.0)
Children		
	No	417 (72.4)
	Yes	159 (27.6)
Alcoholism		
	No	477 (82.8)
	Yes	99 (17.2)
Hypertension		
	No	522 (90.6)
	Yes	54 (9.4)
BMI (categorized) †		
	Underweight/Normal	339 (59.8)
	Overweight	189 (33.3)
	Obesity	39 (6.9)
Seeking mental health help		
	No	530 (92.0)
	Yes	46 (8.0)
Trust in government to handle COVID-19		
	Yes	310 (53.8)
	No	266 (46.2)
Food insecurity		
	No	296 (51.4)
	Yes	280 (48.6)
Physical activity		
	Low	64 (11.1)
	Moderate	39 (6.8)
	High	473 (82.1)
Fear of COVID-19 †		
	No	424 (80.8)
	Yes	101 (19.2)
Work time †		
	1 to 6 months	146 (25.9)
	7 to 12 months	89 (15.8)
	13 to 18 months	123 (21.9)
	19 months or more	205 (36.4)
Emotional exhaustion		
	No	517 (89.8)
	Yes	59 (10.2)
Depersonalization		
	No	351 (60.9)
	Yes	225 (39.1)
Personal realization		
	No	333 (57.8)
	Yes	243 (42.2)
Burnout Syndrome		
	No	524 (91.0)
	Yes	52 (9.0)

* Median (25th percentile–75th percentile). † The sum of the variables is not 576 due to missing values.

**Table 2:** Characteristics associated with burnout syndrome in the military.

Variables		*Burnout Syndrome*		*p* *
		No (n = 524)	Yes (n = 52)	
		n (%)	n (%)	
Age (years) ***		22 (19–31.5)	20 (19–35)	0.422 **
Gender				0.607
	Female	29 (93.6)	2 (6.5)	
	Male	495 (90.8)	50 (9.2)	
Marital status				0.329
	Single	384 (91.4)	36 (8.6)	
	Married	123 (90.4)	13 (9.6)	
	Cohabitant	12 (92.3)	1 (7.7)	
	Divorced	5 (71.4)	2 (28.6)	
Religion				0.951
	None	76 (91.6)	7 (8.4)	
	Catholic	365 (91.0)	36 (9.0)	
	Non-Catholic	83 (90.2)	9 (9.8)	
Children				0.834
	No	380 (91.1)	37 (8.9)	
	Yes	144 (90.6)	15 (9.4)	
Alcoholism				0.057
	No	429 (89.9)	48 (10.1)	
	Yes	95 (96.0)	4 (4.0)	
Hypertension				0.350
	No	473 (90.6)	49 (9.4)	
	Yes	51 (94.4)	3 (5.6)	
BMI (categorized)				0.956
	Underweight/Normal	308 (90.9)	31 (9.1)	
	Overweight	172 (91.0)	17 (9.0)	
	Obesity	36 (92.3)	3 (7.7)	
Seeking mental health help				0.536
	No	481 (90.8)	49 (9.3)	
	Yes	43 (93.5)	3 (6.5)	
Trust in government to handle COVID-19				0.079
	Yes	276 (89.0)	34 (11.0)	
	No	248 (93.2)	18 (6.8)	
Food insecurity				0.213
	No	265 (89.5)	31 (10.5)	
	Yes	259 (92.5)	21 (7.5)	
Physical activity				**0.021**
	Low	63 (98.4)	1 (1.6)	
	Moderate	38 (97.4)	1 (2.6)	
	High	423 (89.4)	50 (10.6)	
Fear of COVID-19				0.072
	No	378 (89.2)	46 (10.9)	
	Yes	96 (95.1)	5 (5.0)	
Time of work				**0.006**
	1 to 6 months	135 (92.5)	11 (7.5)	
	7 to 12 months	86 (96.6)	3 (3.4)	
	13 to 18 months	115 (93.5)	8 (6.5)	
	19 months or more	175 (85.4)	30 (14.6)	

* *p*-value of categorical variables calculated with Chi-Square test.; ** *p*-value of categorical-numerical variables calculated with the U-test (Mann–Whitney).; *** Median—interquartile range.

**Table 3:** Factors associated with burnout syndrome and its dimensions.

Characteristics		*Burnout Syndrome*						*Emotional Exhaustion*						*Depersonalization*						*Personal Realization*					
		Simple Regression			Multiple Regression			Simple Regression			Multiple Regression			Simple Regression			Multiple Regression			Simple Regression			Multiple Regression		
		PR	CI 95%	*p* *	PR	CI 95%	*p* *	PR	CI 95%	*p* *	PR	CI 95%	*p* *	PR	CI 95%	*p* *	PR	CI 95%	*p* *	PR	CI 95%	*p* *	PR	CI 95%	*p* *
Age (years)		1.00	0.97–1.03	0.871	1.00	0.95–1.04	0.845	0.98	0.95–1-01	0.122	0.99	0.95–1.04	0.808	0.99	0.98–1.00	0.05	1.00	0.98–1.02	0.813	1.01	1.00–1.02	0.062	1.00	0.98–1.02	0.927
Gender																									
	Female	Ref.			Ref.			Ref.			Ref.			Ref.			Ref.			Ref.			Ref.		
	Male	1.42	0.36–5.58	0.614	1.26	0.35–4.57	0.727	1.07	0.35–3.22	0.91	0.69	0.17–2.79	0.604	1.01	0.64–1.60	0.954	1.16	0.3–2.15	0.631	0.72	0.52–1.01	0.055	0.78	0.54–1.13	0.192
Marital status																									
	Single	Ref.			Ref.			Ref.			Ref.			Ref.			Ref.			Ref.			Ref.		
	Married	1.12	0.61–2.04	0.724	1.65	0.54–5.09	0.382	0.51.	0.25–1.04	0.065	0.40	0.10–1.70	0.216	0.67	0.50–0.89	0.006	0.53	0.31–0.90	0.020	1.27	1.03–1.56	**0.026**	1.29	0.84–1.98	0.238
	Cohabitant	0.90	0.13–6.01	0.912	1.33	0.27–6.52	0.724	0.66	0.10–4.41	0.666	0.55	0.09–3.38	0.518	0.72	0.32–1.64	0.431	0.44	0.13–1.51	0.189	1.58	1.01–2.48	**0.044**	1.82	1.13–2.94	**0.014**
	Divorced	3.33	0.99–11.22	0.052	8.08	2.05–31.82	**0.003**	1.22	0.19–7.65	0.831	0.58	0.10–3.26	0.539	1.00	0.42–2.34	0.999	0.48	0.16–1.49	0.204	1.10	0.46–2.62	0.824	1.33	0.58–3.07	0.503
Religion																									
	None	Ref.			Ref			Ref.			Ref.			Ref.			Ref.			Ref.			Ref.		
	Catholic	1.06	0.49–2.31	0.874	1.07	0.51–2.21	0.864	0.65	0.35–1.19	0.161	0.58	0.31–1.11	0.099	1.21	0.87–1.69	0.253	1.22	0.87–1.71	0.253	1.72	1.18–2.49	**0.005**	1.55	1.08–2.22	**0.017**
	Non-Catholic	1.16	0.45–2.98	0.758	1.33	0.55–3.24	0.525	0.67	0.30–1.51	0.331	0.41	0.17–0.98	**0.044**	1.35	0.92–1.99	0.128	1.21	0.81–1.79	0.352	1.52	0.98–2.35	0.061	1.44	0.94–2.20	0.092
Children																									
	No	Ref.			Ref.			Ref.			Ref.			Ref.			Ref.			Ref.			Ref.		
	Yes	1.06	0.60–1.88	0.834	0.68	0.27–1.72	0.413	0.74	0.41–1.34	0.325	1.51	0.49–4.64	0.472	0.77	0.59–0.99	**0.038**	1.15	0.72–1.84	0.559	1.28	1.05–1.56	**0.015**	0.89	0.60–1.32	0.563
Alcoholism																									
	No	Ref.			Ref.			Ref.			Ref.			Ref.			Ref.			Ref.			Ref.		
	Yes	0.40	0.15–1.09	0.073	0.40	0.14–1.16	0.092	1.51	0.86–2.64	0.148	1.27	0.72–2.22	0.405	1.23	0.97–1.57	0.089	1.17	0.90–1.53	0.242	0.99	0.76–1.28	0.917	0.95	0.73–1.25	0.721
Hypertension																									
	No	Ref.			Ref.			Ref.			Ref.			Ref.			Ref.			Ref.			Ref.		
	Yes	0.59	0.19–1.84	0.364	0.72	0.25–2.12	0.552	1.09	0.49–2.42	0.832	0.96	0.41–2.25	0.927	0.94	0.65–1.35	0.737	0.85	0.58–1.24	0.405	0.61	0.38–0.96	**0.034**	0.75	0.49–1.15	0.189
BMI (categorized)																									
	Underweight/Normal	Ref.			Ref.			Ref.			Ref.			Ref.			Ref.			Ref.			Ref.		
	Overweight	0.98	0.56–1.73	0.954	0.76	0.38–1.52	0.438	1.12	0.66–1.91	0.666	1.62	0.84–3.14	0.151	0.79	0.62–1.00	0.053	0.86	0.66–1.12	0.259	1.07	0.87–1.31	0.532	0.92	0.72–1.18	0.524
	Obesity	0.84	0.27–2.63	0.766	0.51	0.11–2.44	0.401	1.35	0.56–3.27	0.501	3.21	1.17–8.80	**0.023**	0.97	0.65–1.45	0.895	1.22	0.78–1.90	0.389	1.07	0.73–1.56	0.736	0.98	0.65–1.46	0.909
Seeking mental health help																									
	No	Ref.			Ref.			Ref.			Ref.			Ref.			Ref.			Ref.			Ref.		
	Yes	0.71	0.23–2.18	0.544	0.78	0.26–2.30	0.654	2.07	1.09–3.93	**0.027**	1.81	0.95–3.43	0.07	1.18	0.85–1.65	0.327	1.13	0.81–1.59	0.471	1.10	0.79–1.53	0.578	0.98	0.69–1.39	0.913
Trust in government to handle COVID-19																									
	Yes	Ref.			Ref.			Ref.			Ref.			Ref.			Ref.			Ref.			Ref.		
	No	0.62	0.36–1.07	0.084	0.88	0.51–1.53	0.650	0.80	0.49–1.31	0.374	0.77	0.44–1.34	0.353	1.14	0.93–1.39	0.222	1.08	0.87–1.34	0.497	0.82	0.67–1.00	0.052	0.82	0.67–1.01	0.063
Food insecurity																									
	No	Ref.			Ref.			Ref.			Ref.			Ref.			Ref.			Ref.			Ref.		
	Yes	0.72	0.42–1.22	0.217	0.66	0.39–1.10	0.110	1.44	0.88–2.35	0.146	1.43	0.88–2.32	0.153	1.30	1.06–1.59	**0.013**	1.29	1.03–1.61	**0.024**	1.02	0.84–1.24	0.847	0.99	0.81–1.21	0.918
Physical activity																									
	Low	Ref.			Ref.			Ref.			Ref.			Ref.			Ref.			Ref.			Ref.		
	Moderate	1.64	0.11–25.55	0.724	1.68	0.10–27.46	0.717	0.73	0.24–2.21	0.577	0.98	0.37–2.62	0.968	1.20	0.77–1.86	0.416	1.23	0.80–1.87	0.347	1.52	0.81–2.90	0.198	1.65	0.85–3.21	0.140
	High	6.77	0.95–48.22	0.056	7.15	0.86–59.52	0.069	0.69	0.36–1.35	0.283	0.71	0.34–1.46	0.348	0.94	0.68–1.29	0.706	1.02	0.75–1.39	0.904	2.07	1.29–3.33	**0.003**	2.24	1.34–3.73	**0.002**
Fear of COVID-19																									
	No	Ref.			Ref.			Ref.			Ref.			Ref.			Ref.			Ref.			Ref.		
	Yes	0.46	0.19–1.12	0.087	0.59	0.25–1.37	0.221	5.25	3.21–8.58	**<0.001**	5.71	3.43–9.50	**<0.001**	2.16	1.78–2.62	**<0.001**	2.03	1.66–2.48	**<0.001**	1.16	0.92–1.46	0.221	1.19	0.94–1.52	0.147
Time of work																									
	1 to 6 months	Ref.			Ref.			Ref.			Ref.			Ref.			Ref.			Ref.			Ref.		
	7 to 12 months	0.45	0.13–1.56	0.207	0.48	0.14–1.65	0.246	1.04	0.53–2.03	0.918	0.97	0.48–2.00	0.936	1.11	0.83–1.49	0.497	1.13	0.84–1.53	0.428	1.08	0.77–1.52	0.669	1.01	0.71–1.43	0.956
	13 to 18 months	0.86	0.36–2.08	0.743	0.95	0.36–2.45	0.908	0.88	0.46–1.69	0.703	0.74	0.38–1.42	0.366	1.16	0.89–1.51	0.271	1.15	0.86–1.53	0.351	1.09	0.80–1.48	0.594	1.07	0.76–1.49	0.707
	19 months or more	1.94	1.01–3.75	**0.048**	2.04	1.02–4.10	**0.044**	0.45	0.23–0.90	**0.024**	0.46	0.22–0.97	**0.041**	0.70	0.52–0.93	**0.013**	0.80	0.58–1.10	0.165	1.39	1.08–1.80	**0.011**	1.27	0.95–1.69	0.104

* *p*-values obtained with Generalized Linear Models (GLM), Poisson family, log-link function, robust variance.

## Data Availability

The dataset generated and analyzed during the current study is not publicly available because the ethics committee has not provided permission/authorization to publicly share the data but are available from the corresponding author on reasonable request.
